# *Paenibacillus amylolyticus* 27C64 has a diverse set of carbohydrate-active enzymes and complete pectin deconstruction system

**DOI:** 10.1007/s10295-018-2098-1

**Published:** 2018-10-30

**Authors:** Christian Keggi, Joy Doran-Peterson

**Affiliations:** Department of Microbiology, University of Georgia, 120 Cedar St, 30602 Athens, GA USA

**Keywords:** *Paenibacillus amylolyticus* 27C64, Pectinase, Pectinolytic, CAZymes, Genome

## Abstract

A draft genome of *Paenibacillus amylolyticus* 27C64 was assembled and a total of 314 putative CAZymes in 108 different families were identified. Comparison to well-studied polysaccharide-degrading organisms revealed that *P. amylolyticus* 27C64 has as many or more putative CAZymes than most of these organisms. Four different pectic substrates and xylan supported growth but cellulose was not utilized. Measurement of enzyme activities in culture supernatants revealed low levels of cellulase activity, high levels of xylanase activity, and pectinase activities that adapted to the specific polysaccharides provided. Relative expression levels of each putative pectinase in cells grown with and without three different pectic substrates were evaluated with RT-qPCR and distinct sets of genes upregulated in response to homogalacturonan, methylated homogalacturonan, and rhamnogalacturonan I were identified. It is also noted that this organism’s pectinolytic system differs from other well-studied systems and contains enzymes which are of value for further study.

**Electronic supplementary material:**

The online version of this article (10.1007/s10295-018-2098-1) contains supplementary material, which is available to authorized users.

## Introduction

Plant cell wall polysaccharides have the potential to be a cheap and ubiquitous carbon source for the production of biofuels and chemicals. Pectin is the most structurally complex of these cell wall polysaccharides and is present in all parts of the cell wall but is most prevalent in the outermost layers [33]. The two most abundant types of pectin are homogalacturonan (HG) and rhamnogalacturonan I (RG-I). HG is comprised of an α-1,4 linked galacturonic acid (GalA) backbone that may be methylated (sometimes extensively) or acetylated but does not have side chains. In contrast, RG-I has a backbone of GalA–rhamnose (Rha) disaccharide repeats, has variable arabinose and galactose-rich side chains, and may also be acetylated. Other types of pectic polysaccharides are less abundant than HG and RG-I but are based on the HG-type backbone. For brevity, a full description of these complex polysaccharides is omitted here but has been reviewed in detail elsewhere [33]. The enzymes which deconstruct these polysaccharides, collectively referred to as pectinases, are just as complex as the substrates they work on and are found in all domains of life. Pectinases are broadly divided into two groups: esterases and depolymerases. Esterases remove methyl and acetyl groups decorating the backbone while depolymerases break glycosidic bonds. Depolymerases are further divided based on whether they rely on a hydrolytic mechanism (hydrolases) or β-elimination (lyases), their substrate specificity, and primary products [22]. These enzymes have numerous applications. The use of acidic fungal pectinases to improve fruit juice extraction yields and to clarify the juice is well established [22, 24]. Alkaline pectinases can be used to process plant fibers for the textiles industry, to improve paper production by acting as a biobleaching agent, or to reduce the cationic demand of paper pulp. These alkaline enzymes are also useful in various food processes such as fermenting coffee beans and tea leaves or extracting certain vegetable oils [20, 25].

Of particular interest is the use of pectinases for the production of biofuels and chemicals. Concerns over using edible crops for fuel production, land-use changes from energy crop production, and challenges associated with using woody feedstocks such as the production of compounds inhibitory to fermenting organisms [47] make the use of minimally lignified agricultural waste products attractive. Many such waste products including sugar beet pulp, citrus pulp and peels, and apple pomace are viable feedstocks for ethanol production but are pectin rich and require pectinases for efficient saccharification [17].

Despite the associated challenges, the use of low-pectin lignocellulosic energy crops such as poplar and switchgrass will likely still be necessary for large-scale biofuel production. Unfortunately, efficient and cost-effective enzymatic saccharification of lignocellulosic substrates to fermentable monosaccharides remains a key challenge to their widespread use. In recent years, substantial progress has been made in improving biochemical conversion of lignocellulosic substrates but most of this work has focused on understanding the roles that cellulose, hemicelluloses, and lignin play in biomass recalcitrance [11]. The role of pectin has been largely ignored because of its lower abundance in these substrates. However, recent work has demonstrated that pectin also plays a key role in the recalcitrance of lignocellulosic substrates. For example, expression of enzymes which reduce the amount of demethylated homogalacturonan (HG) in *Arabidopsis thaliana*, tobacco, or wheat improved saccharification of these plants and reduced the need for pretreatment [27, 44]. Improved saccharification has also been demonstrated in woody aspen biomass when a native pectate lyase was overexpressed [6]. Likewise, knockouts or knockdowns of genes involved in pectin biosynthesis in *A. thaliana* [19] or *Populus deltoides* [5] have improved saccharification efficiency. Modification of a biocatalyst has also demonstrated the importance of pectin to biomass recalcitrance: deletion of a pectinase gene cluster in *Caldicellulosiruptor bescii,* a thermophilic anaerobe capable of growth on unpretreated biomass, results in a growth defect on whole plant substrates [12]. This recent evidence that pectin is a barrier to efficient saccharification agrees with older work showing that pectinases are important virulence factors for plant pathogens [13].

Despite these developments, there is still much to learn about pectinases. Although broadly distributed across all domains of the tree of life, subsets of pectinases like those associated with plant pathogens [1], involved in deconstruction of plant material in the human gut [30, 34], and fungal polygalacturonases useful for fruit juice clarification [24] have been studied intensively, and the full diversity of pectin deconstructing enzymes has yet to be explored. In particular, RG-I deconstruction is less well studied than HG deconstruction. A 2015 survey of RG-I deconstructing enzymes was only able to identify 23 characterized examples in the literature [42]. New catalytic activities responsible for pectin deconstruction are still being discovered including one family established in 2006 with the discovery of two novel enzymes from *Bacillus subtilis* [21] and more recently the discovery of several novel enzymes responsible for rhamnogalacturonan II (RG-II) deconstruction in *Bacteroides thetaiotaomicron* [34]. An examination of sequences in the Carbohydrate-Active Enzyme Database (CAZy, http://www.cazy.org/) [29] reveals that while some pectinase families (GH28, PL1) have thousands of sequences identified and more than a hundred listed as characterized, the majority of the remaining families (PL2, PL9, PL10, PL11, PL22, GH105) have ten or fewer characterized sequences listed (PL3 has 24). Even in the well-studied GH28 family, a recent article has highlighted the fact that most of these sequences belong to the genus *Aspergillus* or family *Enterobacteriaceae* and highlights the need to study a more diverse set [26].

One organism with the potential to contribute to understanding of pectinolytic enzymes is *Paenibacillus amylolyticus* 27C64. Originally isolated from the hindgut of an aquatic cranefly (*Tipula abdominalis*) larvae, it is a member of a microbial consortium responsible for the saccharification of conditioned leaf litter [41]. Of the organisms isolated from this gut system, this Gram-positive bacterium displayed the widest array of plant cell wall deconstruction capabilities including cellulose, hemicellulose, and pectinase activities [15]. Two pectate lyases identified through a genomic library screen, PelA and PelB, have demonstrated broad substrate specificities [7] and PelB was able to replace a commercial pectinase mixture in fermentations of pectin-rich cull peaches [18]. Additionally, *P. amylolyticus* is taxonomically divergent from phylogenetic groups whose pectinolytic systems have been studied in detail.

## Methods

### Media and culture conditions

*Paenibacillus amylolyticus* was grown aerobically in tryptic soy broth without dextrose (TSB; 17 g pancreatic digest of casein, 3 g enzymatic digest of soybean meal, 5 g NaCl, 2.5 g dipotassium phosphate per liter) at 37 °C with shaking unless stated otherwise. Tryptic soy agar (15 g pancreatic digest of casein, 5 g papaic digest of soybean meal, 5 g NaCl, 15 g agar per liter) was used for solid media. All media were purchased from Becton–Dickinson (Franklin Lakes, NJ). Polygalacturonic acid (PGA), citrus pectin, apple pectin, galacturonic acid (GalA), glucose, crystalline cellulose (Sigmacell), oat spelt xylan, and CaCl_2_ were purchased from Sigma-Aldrich (St. Louis, MO). RG-I from soy was purchased from Megazyme (Bray, County Wicklow, Ireland).

### Genome sequencing, assembly, and annotation

Total genomic DNA was isolated from *P. amylolyticus* 27C64 using a Promega Wizard Genomic DNA Purification Kit (Madison, WI). Library preparation and sequencing was performed by the Georgia Genomics Facility (Athens, GA) using the Illumina NextSeq 500 platform (San Diego, CA) with paired-end 150 base pair reads. The reads were quality trimmed using Trimmomatic [8] and assembled with the SPAdes assembler [4] by the Quantitative Biology Consulting Group (QBCG) at the University of Georgia (Athens, GA). The draft genome was filtered to exclude contigs with an average read coverage less than two or which returned no hits in a BLAST + search against NCBI’s non-redundant nucleotide database [9]. The assembled draft genome was annotated with Rapid Annotation using Subsystem Technology (RAST) [3, 38].

Pairwise average nucleotide identity (ANI) between this draft genome and every other *Paenibacillus* genome available through the Joint Genome Institute’s Integrated Microbial Genomics (IMG) website (https://img.jgi.doe.gov/) [10] was calculated using their pairwise ANI tool [46].

### Carbohydrate-active enzyme identification and annotation

Carbohydrate-active enzymes (CAZymes) were identified using two tools: the CAZy Analysis Toolkit (CAT) [39] and the Database for Carbohydrate-Active Enzyme Annotation (dbCAN) [48]. Both tools identify CAZymes and assign them to a family in the Carbohydrate-Active Enzyme (CAZy; http://www.cazy.org/) database [29].

To identify putative pectinases, an E-value cutoff of 0.01 was used for CAT with default cutoffs for dbCAN and the merged results were filtered to exclude all CAZy families not known to contain pectinases. Genes on this narrowed list were manually reviewed based on evidence from BLAST + searches [9] against the CAZy database and NCBI non-redundant protein database, by evaluating conserved protein domains with CD-Search [32], and by performing multiple sequence alignments against characterized members of each CAZy family using Multiple Sequence Comparison by Log-Expectation (MUSCLE) [16]. Initial putative functions were assigned based on the list of known activities in the CAZy family to which they belong. Subcellular localization of each putative pectinase was predicted with SignalP 4.0 [40], PSORTb [49], and Phobius [23].

Only dbCAN results were used to generate a summary of CAZymes in the genome because of the relatively low false-positive rate. Genes that did not match a catalytic domain and had only a carbohydrate binding domain (CBM) or S-layer homology domain (SLH) were excluded. Any hits to CE10 family were also excluded since this family is now defunct.

### Growth curves

TSB was made as per the manufacturer’s instructions and MOPS minimal media was made as previously described [35]. Both types of media were supplemented with a final concentration of 0.1 mM CaCl_2_, adjusted to a final pH of 7.0, and filter sterilized through a 0.22-µM PES membrane (Stericup, EMD Millipore, Burlington, MA). All carbohydrates were added to a final concentration of 0.4% w/v, the substrate concentration that was identified as optimal for PGA. For growth curves with soluble pectic substrates, five milliliters of each medium evaluated was inoculated to an OD_600_ of 0.01 with a washed *P. amylolyticus* cell suspension. Cell suspensions were prepared by pelleting 1 mL of each of four overnight cultures, washing once in sterile saline, and resuspending in 500 µL of saline. The OD_600_ of each resuspension was measured in duplicate. Three hundred microliters of inoculated media were dispensed into each of nine wells with one uninoculated blank well and growth at 37 °C with constant maximal shaking was monitored at 600 nm using a Bioscreen C (Growth Curves USA, Piscataway, NJ). Growth in each well was normalized with the appropriate blank, measurements of technical replicates were averaged, and mean OD and standard deviation were calculated for the four biological replicates. After growth, technical replicates were pooled, mixed, pelleted, and cell-free supernatant was harvested for enzyme assays.

Media with insoluble substrates (cellulose and xylan) were prepared by autoclaving the appropriate amount of each substrate in 12.5 mL of distilled water in a 125-mL shake flask then adding 12.5 mL of 2X TSB or 2X MOPS minimal media prepared as for the soluble substrates but in half the final volume. Inoculation to an initial OD of 0.01 was performed exactly as above. Growth was monitored by spread plating cells diluted into sterile saline onto TSA. Colonies were counted after incubating overnight at 37 °C.

### Measurement of enzyme activities

Pectate lyase activity was measured as previously described [14]. Briefly, 5 µL of culture supernatant was added to 1 mL of substrate solution (100 mM Tris–HCl, pH 8.5, 0.2% w/v PGA, 0.5 mM CaCl_2_) which had been pre-equilibrated to 25 °C and mixed. The absorbance at 232 nm was monitored for 2 min and the slope was used with the published molar extinction coefficient to calculate product formation. Activity is reported in international units of enzyme activity (IU) per mL (1 unit = 1 µmol product formed in 1 min). Pectin lyase assays were conducted similarly but with a different substrate solution (100 mM Tris–HCl, pH 8.0, 0.2% w/v 85% methoxy citrus pectin, 1 mM EDTA) as previously described [14]. Rhamnogalacturonan lyase activity was measured with this assay as well using a RG-I substrate solution (50 mM Tris–HCl, pH 7.5, 0.05% w/v RG-I from potato, 2 mM CaCl_2_) and one unit of activity was defined as the amount of enzyme that increased absorbance by 1.0 in 1 min [37]. RG-I from potato was purchased from Megazyme (County Wicklow, Ireland) and 85% methoxy citrus pectin was from Sigma (St. Louis, MO).

Xylanase activity was measured using a miniaturized version of the Nelson–Somogyi reducing sugar assay with the standard copper and arsenomolybdate reagents [36, 43]. For each assay, 50 µL of xylan substrate solution (2% w/v oat spelt xylan solubilized overnight at 37 °C with shaking in 20 mL of 0.2 N NaOH, 100 mM MOPS, pH 7, 0.02% w/v sodium azide) was mixed with 50 µL of culture supernatant and incubated for 15 min at 40 °C. Reactions were stopped with 200 µL of copper reagent, boiled for 10 min, cooled, and 200 µL of color reagent was added. Samples were diluted fivefold and the absorbance was measured at 500 nm. Sugar release was determined relative to a xylose standard curve. Enzyme activity was calculated in IU/mL after subtracting the values of substrate only and supernatant only blanks for each sample. Oat spelt xylan was purchased from Sigma (St. Louis, MO).

Cellulase activity was measured similarly except a carboxymethyl cellulose (CMC) substrate solution (1% w/v CMC, 100 mM MOPS, pH 7, 0.02% w/v sodium azide) was used, reactions were allowed to proceed for 4 h, and glucose was used as the standard. CMC was purchased from Sigma (St. Louis, MO).

### RT-qPCR

Total RNA was isolated from cells grown in TSB without pectin and in TSB supplemented with 0.4% w/v of either PGA, apple pectin, or RG-I from soy at 12.75 h after inoculation with the ZR Fungal/Bacterial RNA Miniprep kit (Zymo Research, Irvine, CA). Ten µg of RNA was treated with DNase using the TURBO DNA-free Kit (Thermo Fisher, Waltham, MA). RNA was used as a PCR template to confirm that no genomic DNA was remaining. One microgram of each sample was reverse transcribed with the iScript cDNA Synthesis Kit (Bio-Rad, Hercules, CA) and diluted 1:10 with nuclease-free water. Two microliter of diluted cDNA was used as template in 20-µL qPCRs with Luna Universal qPCR Master Mix (New England Biolabs, Ipswich, MA) and run on a StepOne Plus instrument (Applied Biosystems, Foster City, CA). All conditions were repeated with four biological replicates. Oligonucleotide primers were purchased from Integrated DNA Technologies (Coralville, IA).

Six potential reference genes (*ftsZ*, *rpoD*, *era*, *adk*, *gyrA*, and *gap* homologues) were evaluated and ranked using the geNorm algorithm [45] and two most stable genes, *ftsZ* and *rpoD*, were selected as endogenous control genes. Quantification thresholds and Cq values were calculated by the StepOne Plus software. Fold change in expression was calculated using the 2^−∆∆Cq^ method [28].

## Results and discussion

### Genome sequencing, assembly, and annotation

The draft genome consisted of 103 contigs totaling 6.77 Mb in length with 5922 protein coding sequences and 93-fold average read coverage. The 20 largest contigs accounted for > 90% of the total genome length while the largest 33 contigs account for 99% of the genome length (N50 = 325,177 bp, L50 = 7). The remaining small contigs accounting for ~ 1% of genome length were composed primarily of repetitive rRNA and tRNA elements.

Pairwise average nucleotide identity comparison of this draft genome against all 217 public *Paenibacillus* genomes available through IMG revealed no other genome above the recommended species cutoff (ANI ≥ 96.5 and AF ≥ 0.6) [46]. The most closely related genome, *Paenibacillus phyllostachyicola* BL9 (*Paenibacillus polysaccharolyticus* BL9T) [31], had an ANI of 93.2% with an AF of 0.84. The ANI of the next most related genome, *Paenibacillus sp.* PAMC 26,794, was much lower at 79.99% ANI and an AF value of 0.72. Interestingly, the only *P. amylolyticus* genome already published, *P. amylolyticus* Heshi-A3 [2], was the eighth most related with an ANI value of 79.82 and an AF of 0.70, well below the recommended speciation cutoff. These data suggest that this isolate is not the same species as any of the other IMG publicly available genomes.

### CAZyme identification and annotation

**Fig. 1:**
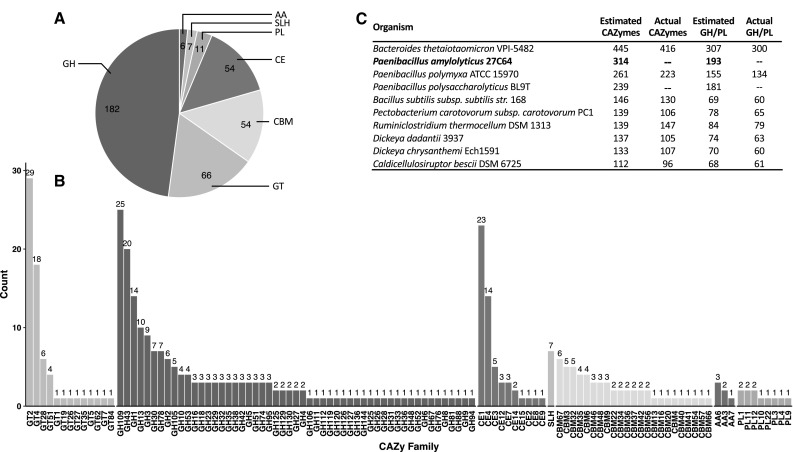
Summary of dbCAN results. All predicted protein sequences from *P. amylolyticus* 27C64 were submitted to the dbCAN webserver and compared to hidden Markov models of each CAZy family. Hits below the published cutoffs were excluded. Hits are summarized by CAZy catalytic activity (**a**) and CAZy family (**b**). Identical analyses were carried out on other bacterial genomes for comparison (**c**). The dbCAN results are presented in the “Estimated” columns while the number of CAZymes recognized by the CAZy database is presented as “Actual” columns for those organisms present in the database

Analysis of the whole genome for CAZymes revealed that 314 (5.3%) of the 5922 predicted coding sequences matched one or more CAZy families. These hits included matches to all five CAZy catalytic groups as well as to CBM domains with glycoside hydrolases being the most represented group (Fig. 1a). These genes represented 110 different CAZy families including CBMs, 87 of which are catalytic families (Fig. 1b). A total of 193 glycoside hydrolases (GH) or polysaccharide lyases (PL), catalytic activities responsible for deconstructing polysaccharides, were identified in 53 different GH families and 8 PL families (Fig. 1b). Seven SLH domains, sometimes important for CAZyme attachment to the cell surface, are not a CAZy family but were also identified by dbCAN. Although the exact number of false positives or unidentified CAZymes within these results cannot be determined precisely, it has been previously demonstrated that dbCAN correctly identifies 99.3% of the true CAZymes in the well-studied *C. thermocellum* genome and that 89.4% of hits generated were true CAZymes [48]. A majority of the hits generated by dbCAN in this genome were very good matches: 227 or 72.3% of these 314 putative CAZymes had an *E* value less than 1 × 10^−20^ and 138 or 44.0% had an *E* value less than 1 × 10^−50^. This analysis validated previous culture-based work which showed that *P. amylolyticus* 27C64 has a diverse array of polysaccharide deconstruction capabilities [15]. In fact, *P. amylolyticus* 27C64 has two to three times as many predicted GH and PL enzymes as cellulolytic thermophiles such as *Caldicellulosiruptor bescii* and *Ruminiclostridium thermocellum* (*Clostridium thermocellum*) which have been of interest for the conversion of lignocellulosic material to biofuels (Fig. 1c). It also has more predicted GH and PL enzymes than well-studied pectin-degrading bacteria (*Dickeya chrysanthemi, Dickeya dadantii, Pectobacterium carotovorum*, and *Bacillus subtilis*). Within its own genus, it tops both *Paenibacillus polymyxa* and the most closely related genome *P. polysaccharolyticus*. Of all the bacterial genomes selected for comparison, only the human gut-associated bacterium *Bacteroides thetaiotaomicron* had more predicted GH and PL enzymes.

To evaluate the extent to which the richness of putative CAZymes in the genome might be related to the deconstruction of plant cell wall polysaccharides specifically, the presence of putative cellulases, xylanases, and pectinases was examined. After removing any RAST hits to cellulases that were not assigned to a CAZy family by dbCAN, a total of three putative endoglucanases and six putative β-glucosidases were identified. Similarly, 17 endo-xylanases, six β-xylosidases, and two acetyl xylan esterases were identified. Pectinases were difficult to identify in the same manner because of their diversity, inaccurate assignment by RAST, and inconsistent naming in databases. Instead, hits to CAZy families relevant to pectin deconstruction were manually reviewed to identify 28 putative pectinases including a variety of lyases, hydrolases, and esterases related to both HG and RG-I deconstruction. The presence of numerous putative plant cell wall polysaccharide deconstruction enzymes is consistent with this organism’s role as a member of an insect hindgut community responsible for degrading conditioned leaf litter.

### Growth on plant cell wall polysaccharides

**Fig. 2:**
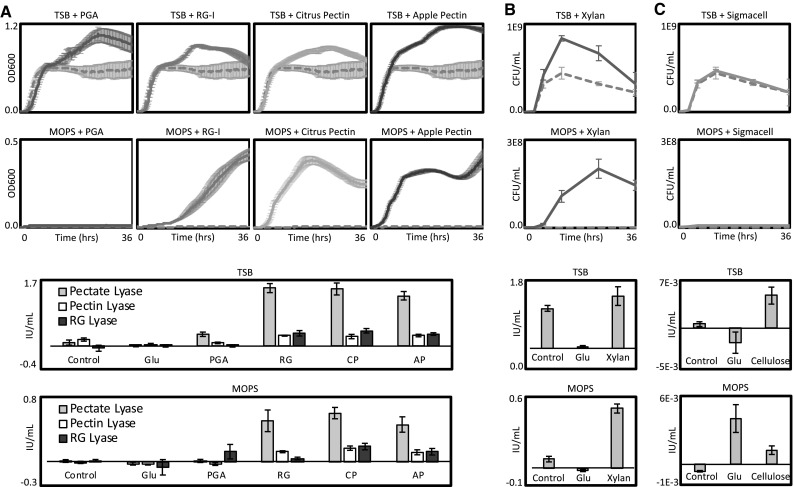
Growth of *P. amylolyticus* 27C64 on four different pectins (**a**), oat spelt xylan (**b**), and crystalline cellulose (**c**). Upper panels depict growth on each substrate as either a supplemental carbon source in TSB without dextrose or as a sole carbon source in MOPS minimal media. Dashed gray lines indicate the control condition (no polysaccharide added). Growth was measured as the OD at 600 nm for soluble substrates and based on plate counts of serial dilutions for insoluble substrates. Lower panels depict the amount of pectinase (**a**), xylanase (**b**), or cellulase (**c**) activity in cell-free supernatants harvested from each growth condition at the end of the growth period. All experiments were conducted with four biological replicates and error bars indicate the standard deviation. *Glu* glucose, *PGA* polygalacturonic acid, *RG* rhamnogalacturonan I, *CP* citrus pectin, *AP* apple pectin

To evaluate whether this CAZyme diversity allowed this organism to use plant cell wall polysaccharides as a carbon source, *P. amylolyticus* was grown with various polysaccharides as a sole and supplemental carbon source (Fig. 2). Improved growth was observed when TSB was supplemented with each of four different pectins (Fig. 1a) and xylan (Fig. 1b) but not when crystalline cellulose was added. Only three of the four pectins could be utilized as a sole carbon source in MOPS minimal media, with PGA failing to support growth. Xylan also supported growth as a sole carbon source but cellulose did not.

At the end of each growth curve, culture supernatant was harvested and assayed for relevant enzymatic activity. Supernatant from TSB or MOPS cultures with glucose was also included in these assays. Since *P. amylolyticus* was unable to utilize cellulose for growth, it was unsurprising that only very low cellulase activity was observed overall. In contrast, strong xylanase activity was observed when *P. amylolyticus* was grown in TSB, in TSB with xylan, and in MOPS with xylan. Glucose completely repressed xylanase production in both media backgrounds.

Similarly, all three pectinase activities measured (pectate lyase, pectin lyase, and RG lyase) were completely repressed by glucose. Pectate lyases, which cleave demethylated HG, were expressed at high levels when RG-I, citrus pectin, or apple pectin were supplied for growth in either TSB or MOPS. The much lower amount of activity observed after growth in TSB with PGA was unexpected since pectate lyases would be primarily responsible for deconstruction of PGA in an organism without extracellular polygalacturonases. This suggests that a pectin degradation product responsible for inducing these pectate lyases is absent from the homogeneous PGA substrate. This low level of induction likely explains the lack of growth on PGA as a sole carbon source. RG lyase activity could be observed when RG-I was supplied for growth or when whole citrus and apple pectins (which include some RG-I) were present. PGA did not induce these enzymes. In contrast to the pectate and RG lyase activities, pectin lyase activity appeared at similar levels in the TSB control condition as it did in all other conditions that allowed for growth except TSB with glucose. Since pectin lyases cleave only methylated pectin, it is possible that this activity is responsible for initial release of methylated fragments from the web of cell wall polysaccharides and is constitutively expressed. It is likely that this activity, along with very low levels of pectate or RG lyase activities, releases the degradation products that induce the rest of the system. The fact that this organism’s deconstruction system adapts to various pectic polysaccharides indicates that different parts of the pectinolytic system are responding to different inducers.

### Differential expression of putative pectinases

The number and diversity of putative pectinases made this system in particular seem of value for further study, but more information was necessary to identify the role of each gene in the overall system. The adaptability of the pectinolytic system to different substrates made it possible to better define the role of each individual element by comparing the relative expression of each gene when cells were grown with and without different pectic polysaccharides. RNA was isolated from cells grown in PGA and RG-I from soy because they should reliably induce the HG and RG-I deconstruction systems specifically. Apple pectin was also included because the HG it contains is partially methylated, unlike PGA.

**Fig. 3:**
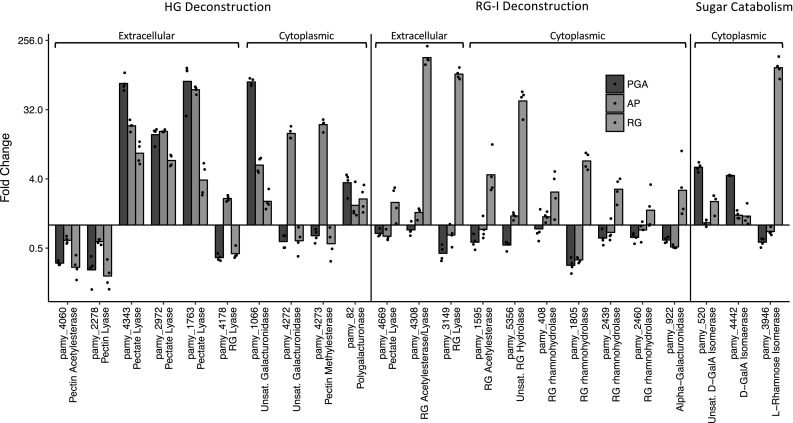
Differential expression of putative pectinases when grown on three different pectic substrates. Points indicate the fold change in expression as compared to the control condition (TSB without pectin) for individual biological replicates and columns indicate the mean fold change values of all four biological replicates. Fold change values were calculated with the ∆∆Ct method using ftsZ and rpoD as reference genes. *HG* homogalacturonan, *RG* rhamnogalacturonan I, *PGA* polygalacturonic acid, *AP* apple pectin

In total, 18 of the 28 putative pectinases were upregulated on one or more pectic substrates (Fig. 3). The pectin lyase, which appeared to be constitutively expressed in the growth experiments, was among the ten genes not upregulated on any substrate but is included in the putative pectinolytic system. So too is the putative pectin acetylesterase which cannot be excluded by RT-qPCR alone since none of the substrates used in this experiment had highly acetylated HG. The other eight genes not upregulated in any condition are likely to have some other activity unrelated to pectin deconstruction (Figure S1). Growth with PGA largely resulted in several expected changes: three extracellular pectate lyases (*pamy_4343, pamy_2972, pamy_1763*) and one cytoplasmic polygalacturonase (*pamy_82*) were upregulated. The isomerases responsible for initiating catabolism of saturated and unsaturated GalA were also upregulated (*pamy_4442, pamy_520*). The increased expression of one GH105 family hydrolase (*pamy_1066*) on PGA suggests that this enzyme specifically removes an unsaturated GalA from the end of HG oligosaccharides instead of from RG-I fragments, an activity which has only recently been demonstrated within that family [30].

Growth on apple pectin resulted in the upregulation of the all the PGA-induced enzymes as well as the putative pectin methylesterase (*pamy_4273*), one RG lyase (*pamy_4178*), and a second GH105 hydrolase (*pamy_4272*). Upregulation of the pectin methylesterase on this substrate was anticipated given the partial methylation of the substrate, but the increased GH105 hydrolase production (which is likely cotranscribed with the pectin methylesterase) suggests that this enzyme is a HG-specific unsaturated galacturonidase like *pamy_1066* but is specific for methylated substrates. Likewise, upregulation of the putative RG lyase *pamy_4178* was unexpected, but the potential utility of this enzyme for deconstructing methylated HG substrates is unclear.

Growth with RG-I resulted in lower upregulation of the PGA-inducible genes, likely because the RG-I substrate is not completely separated from the other pectic polysaccharides. However, this substrate produced high fold change values for expected RG-I deconstructing enzymes including two extracellular RG lyases (*pamy_4308, pamy_3149*) and one additional GH105 hydrolase (*pamy_5356*). Cytoplasmic rhamnosidases (*pamy_408, pamy_1805, pamy_2439, pamy_2460*) were less highly upregulated and it is possible that one or more of these enzymes is not specific to RG-I deconstruction. The one putative cytoplasmic galacturonidase (*pamy_922*) was upregulated by a similar amount. Interestingly, a fourth pectate lyase (*pamy_4669*) which was not upregulated on PGA or apple pectin did appear somewhat upregulated in response to RG-I.

### Summary of the putative pectinolytic system

**Fig. 4:**
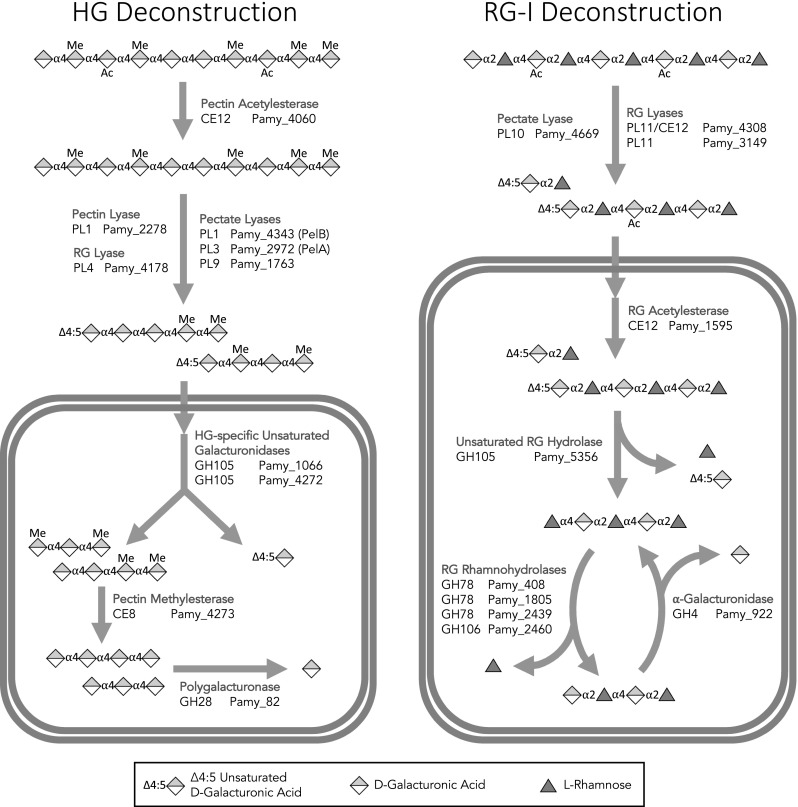
Summary of putative pectinolytic system. Symbols are based on the standardized symbol nomenclature for glycan notation. CAZy family assignments are provided to the left of each gene. *HG* homogalacturonan, *RG-I* rhamnogalacturonan-I, *GalA*d-galacturonic acid, *Rha*l-rhamnose

A summary of the putative pectinolytic enzymes is provided (Table 1) as well as a diagram of the system based on the bioinformatic and RT-qPCR analyses (Fig. 4). Overall this system includes a diverse set of pectinases from 13 different CAZy catalytic families (plus one CBM family) including some of the less well-studied pectate lyase families, the recently described GH105 hydrolases, and both known RG lyase families. In addition, the existence of a GH105 hydrolase specifically upregulated on methylated HG (*pamy_4272*) has not been described in the literature and it is possible that this enzyme has a novel activity. Also, the presence of an intracellular pectin methylesterase (*pamy_*4273) is unusual since demethylation is generally thought of as an early step in deconstruction. This perhaps explains earlier observations that PelA and PelB retain activity on methylated substrates [7]; this flexibility would be necessary in a system which lacks an extracellular methylesterase. It is likely that the other two pectate lyases in this system also share this trait. Identification of additional enzymes with such broad substrate specificities is potentially valuable because they may allow for the creation of simplified pectinase mixtures which are as effective as more complex cocktails.

**Table 1:** Putative pectinases of *P. amylolyticus* 27C64

Gene	CAZy family	Putative assignment	dbCAN E value	Gene length (kb)	Protein size (kDa)	Signal peptide
HG deconstruction						
Pamy_4060	CE12	Pectin acetylesterase	2.00E-52	1.2	41.9	Yes
Pamy_2278	PL1_8	Pectin lyase	5.50E-91	1.1	38.7	Yes
Pamy_4343	PL1	Pectate lyase (PelB)	3.20E-41	1.4	51	Yes
Pamy_2972	PL3_1	Pectate lyase (PelA)	1.10E-72	0.7	23.4	Yes
Pamy_1763	PL9	Pectate lyase	8.20E-135	1.4	47.8	Yes
Pamy_4178	PL4_1	Rhamnogalacturonan lyase	8.20E-199	1.6	57.7	Yes
Pamy_1066	GH105	HG-specific unsaturated galacturonidase	3.50E-121	1.1	42.2	No
Pamy_4272	GH105	HG-specific unsaturated galacturonidase	1.80E-118	1.1	42.1	No
Pamy_4273	CE8	Pectin methylesterase	1.60E-96	1	38.4	No
Pamy_82	GH28	Polygalacturonase	1.70E-78	1.6	58.7	No
RG-I Deconstruction						
Pamy_4669	PL10	Pectate lyase	4.90E-99	1	34.9	Yes
Pamy_4308	PL11|CE12|CBM37	Rhamnogalacturonan lyase/acetylesterase	2.00E-287	5.2	188.2	Yes
Pamy_3149	PL11_1	Rhamnogalacturonan lyase	6.70E-153	2.4	86.1	Yes
Pamy_1595	CE12	Rhamnogalacturonan acetylesterase	4.80E-77	1.3	49.2	No
Pamy_5356	GH105	Unsaturated rhamnogalacturonyl hydrolase	1.90E-78	1	39.2	No
Pamy_408	GH78	Rhamnogalacturonan rhamnohydrolase	1.30E-185	2.7	102.3	No
Pamy_1805	GH78	Rhamnogalacturonan rhamnohydrolase	6.00E-79	1.6	61.7	No
Pamy_2439	GH78	Rhamnogalacturonan rhamnohydrolase	5.90E-172	2.8	106.8	No
Pamy_2460	GH106	Rhamnogalacturonan rhamnohydrolase	2.10E-122	2.8	105.2	No
Pamy_922	GH4	α-Galacturonidase	3.20E-68	1.3	48.7	No

The RG-I deconstruction system also contains unique features. Most notably, the extracellular enzyme *Pamy_4308* contains both RG lyase and RG acetylesterase domains and is likely able to cleave acetylated RG-I directly. A single enzyme with both of these catalytic domains has not yet been described, and RG lyases characterized to date have been inhibited by acetylation [42]. The presence of a putative cytoplasmic RG acetylesterase is unusual for the same reason that the intracellular pectin methylesterase was unexpected and likewise makes sense in the context of an extracellular RG lyase which may act on acetylated substrates. As in the case of the HG deconstruction system, identification of RG depolymerases which are uninhibited by acetylation may help improve and simplify pectinase mixtures.

## Conclusion

*P. amylolyticus* 27C64 is distinct from other published *Paenibacillus* genomes and has a large and diverse set of putative CAZymes putting it on par with well-studied polysaccharide deconstructing organisms. Its pectinolytic system allows it to utilize four different pectic substrates as carbon sources and adapts to the specific substrate available. This system differs from other well-studied systems because it appears to rely on cytoplasmic deesterification of substrates and has enzymes with potentially novel functions and domain architectures. Direct biochemical study of this pectinolytic system may lead to improved understanding of the full diversity of natural pectinolytic systems.

## Electronic supplementary material

Below is the link to the electronic supplementary material.

Supplementary material 1 (PDF 10 kb)
