# Supplementary material for: Paenibacillus amylolyticus 27C64 has a diverse set of carbohydrate-active enzymes and complete pectin deconstruction system
Source: J Ind Microbiol Biotechnol. 2018 Oct 30;46(1):1–11. doi: 10.1007/s10295-018-2098-1 (PMC6339884; doi:10.1007/s10295-018-2098-1)
Supplement: Supplementary file 1 — Supplementary material 1 (PDF 10 kb) [file 10295_2018_2098_MOESM1_ESM.pdf]

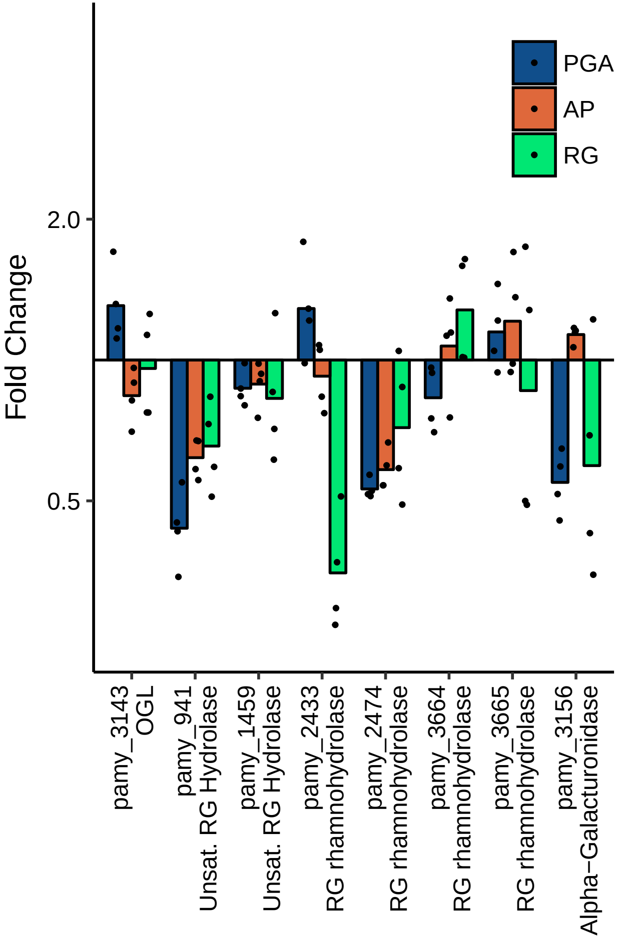
PGA
AP
RG
Fold Change
2.0
0.5
pamy_3143
OGL
pamy_941
Unsat. RG Hydrolase
pamy_1459
Unsat. RG Hydrolase
pamy_2433
RG rhamnohydrolase
pamy_2474
RG rhamnohydrolase
pamy_3664
RG rhamnohydrolase
pamy_3665
RG rhamnohydrolase
pamy_3156
Alpha−Galacturonidase
